# Hyper‐metabolic B cells in the spleens of old mice make antibodies with autoimmune specificities

**DOI:** 10.1186/s12979-021-00222-3

**Published:** 2021-02-27

**Authors:** Daniela Frasca, Maria Romero, Denisse Garcia, Alain Diaz, Bonnie B. Blomberg

**Affiliations:** 1 Department of Microbiology and Immunology, University of Miami Miller School of Medicine, RMSB 3146A, 1600 NW 10th Ave, FL 33136 Miami, USA; 2 Sylvester Comprehensive Cancer Center, University of Miami Miller School of Medicine, Miami, FL USA

**Keywords:** Aging, B cells, Metabolism

## Abstract

**Background:**

Aging is associated with increased intrinsic B cell inflammation, decreased protective antibody responses and increased autoimmune antibody responses. The effects of aging on the metabolic phenotype of B cells and on the metabolic programs that lead to the secretion of protective versus autoimmune antibodies are not known.

**Methods:**

Splenic B cells and the major splenic B cell subsets, Follicular (FO) and Age-associated B cells (ABCs), were isolated from the spleens of young and old mice and left unstimulated. The RNA was collected to measure the expression of markers associated with intrinsic inflammation and autoimmune antibody production by qPCR. B cells and B cell subsets were also stimulated with CpG and supernatants collected after 7 days to measure autoimmune IgG secretion by ELISA. Metabolic measures (oxygen consumption rate, extracellular acidification rate and glucose uptake) were performed using a Seahorse XFp extracellular flux analyzer.

**Results:**

Results have identified the subset of ABCs, whose frequencies and numbers increase with age and represent the most pro-inflammatory B cell subset, as the cell type mainly if not exclusively responsible for the expression of inflammatory markers and for the secretion of autoimmune antibodies in the spleen of old mice. Hyper-inflammatory ABCs from old mice are also hyper-metabolic, as compared to those from young mice and to the subset of FO B cells, a feature needed not only to support their higher expression of RNA for inflammatory markers but also their higher autoimmune antibody secretion.

**Conclusions:**

These results identify a relationship between intrinsic inflammation, metabolism and autoimmune B cells and suggest possible ways to understand cellular mechanisms that lead to the generation of pathogenic B cells, that are hyper-inflammatory and hyper-metabolic, and secrete IgG antibodies with autoimmune specificities.

## Background

Aging is associated with increased low-grade systemic inflammation, called inflammaging [1], that has been associated with decreased protective antibody responses against infections and vaccines. Inflammaging induces intrinsic inflammation in immune cells that leads to functional impairment. Data from our laboratory [2] have shown that unstimulated splenic B cells from old mice make more TNF-α than those from young mice. We have hypothesized and showed that this “pre-activated” phenotype of the B cells from old mice makes them refractory to further stimulation to generate protective antibody responses in both mice [2] and humans [3]. To confirm our hypothesis, we pre-incubated splenic B cells with exogenous TNF-α before stimulation and found that this pre-treatment significantly decreases both young and old B cell responses. Conversely, blocking TNF-α by adding an anti-TNF-α antibody to B cells before stimulation significantly increases class switch in young and more significantly restores it to young levels in old cultured B cells and in vivo [2].

Aging is also associated with metabolic dysfunction. Decreased insulin sensitivity [4, 5] and mitochondrial function [6, 7], as well as dysregulated nutrient uptake [8], have been reported. Inflammaging-induced metabolic dysfunction represents a significant risk factor for morbidity and mortality of elderly individuals as it is implicated in the pathogenesis of several debilitating chronic diseases of old age including type-2 diabetes mellitus [9], osteoporosis [10], Alzheimer’s disease [11], rheumatoid arthritis [12], and coronary heart disease [13].

Immune cell function is critically supported by metabolic pathways. The major pathways utilized to generate energy are: (1) anaerobic glycolysis, in which glucose is incompletely oxidized in the cytosol (glycolysis), yielding lactate as the final product. It is fast but energy inefficient; and (2) oxidative phosphorylation (OXPHOS), in which carbon substrates such as glucose-derived pyruvate, fatty acids and glutamine are oxidized in the mitochondria to generate ATP. The effects of aging on metabolic pathways associated with immune cell function are understudied [14]. Moreover, virtually nothing is known about the metabolic phenotype of B cells and if aging induces changes in the metabolic programs that lead to the secretion of protective versus autoimmune antibodies. The first and only published study on age-related changes in metabolic pathways in murine B cells was performed on antibody-secreting cells (ASCs) isolated from the bone marrow of young and old mice [15]. Results there showed that genes involved in lipid and carbohydrate metabolism were up-regulated in old versus young ASCs, and this hypermetabolic profile of old ASCs was associated with higher expression of PD-1 and cellular ROS, markers of intrinsic B cell inflammation and cell exhaustion, and lower total IgG secretion.

In this paper we evaluated the metabolic profile of B cells isolated from the spleens of young and old mice, with the aim to identify metabolic pathways associated with intrinsic B cell inflammation and with the secretion of autoimmune antibodies. We focused on the secretion of autoimmune antibodies because our recent human B cell results [16] have shown that higher intrinsic inflammation in unstimulated B cells from elderly individuals induces a “pre-activation” status associated with the secretion of IgG antibodies with autoimmune specificities, similar to what has been observed in autoimmune diseases [17], and in the obese adipose tissue at least for some specificities [18, 19]. In order to identify the B cell subset(s) driving the phenotype and function of B cells in the splenic B cell pool of old mice, we sorted the major splenic B cell subsets, Follicular (FO) and Age-associated B cells (ABCs). Results have shown that ABCs are the cells driving the phenotype and function of B cells in the spleen of old mice. Hyper-inflammatory ABCs from old mice are also hyper-metabolic and supported by a specific metabolic profile needed not only to support intrinsic inflammation but also autoimmune antibody secretion.

## Results and discussion

### B cells from old mice, as compared to B cells from young mice, are characterized by higher frequencies of ABCs which are responsible for higher expression of pro-inflammatory markers and of markers associated with autoimmune antibody production

**Fig. 1:**
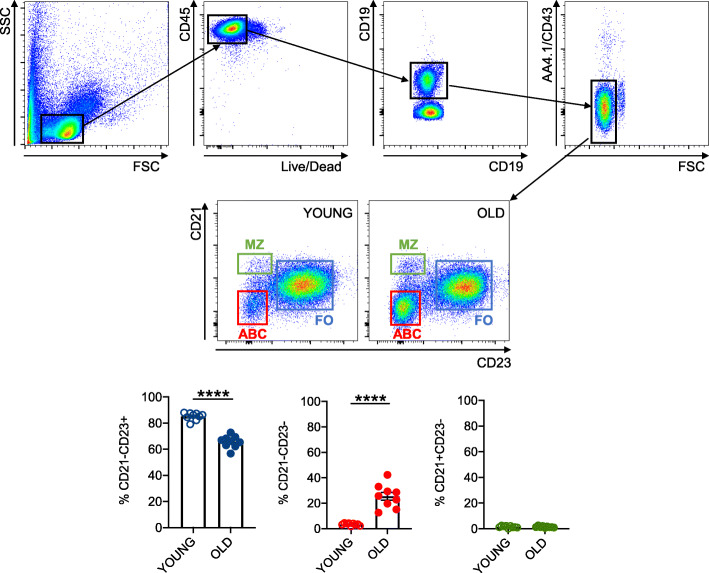
Composition of the splenic B cell pool of young and old mice.The spleens of 9 pairs of young and old mice were stained to evaluate percentages of the major B cell subsets. Results show percentages of FO, ABCs and MZ B cells. **Top**. Gating strategies showing that cells are first gated on Live CD45+ to exclude dead cells and then on CD19+AA4.1-CD43- cells to exclude transitional (AA4.1+) and B1 (CD43+) B cells. **Center**. A representative dot plot of splenic B cells from one young and one old mouse. **Bottom**. Frequencies of FO, ABCs and MZ B cells. Mean comparisons between groups were performed by unpaired Student’s t test (two-tailed). ****p<0.0001

The composition of the splenic B cell pool of the young and old mice in this study is shown in Fig. 1. Briefly, we measured the percentages of Follicular (FO, CD19+AA4.1/CD43-CD23+CD21-), Age-associated B cells (ABCs, CD19+AA4.1/CD43-CD23-CD21-) and Marginal Zone (MZ, CD19+, AA4.1/CD43-CD23-CD21+) B2 B cell subsets in the spleens of young and old mice. Results show that ABC frequency and number increase with age at the expense of FO, whereas no significant changes were observed in the MZ B cell subset as previously shown by us [20, 21] and by other groups [2, 22].

**Fig. 2:**
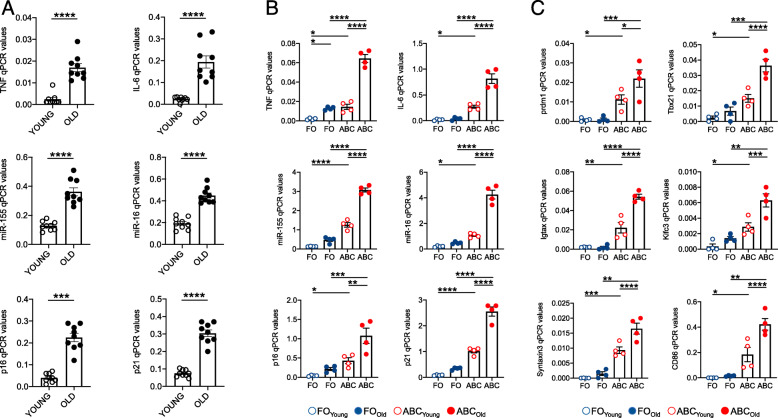
Total B cells and ABCs from old mice express higher levels of RNA for pro-inflammatory markers, and markers of autoimmune antibody production, as compared to those from young mice.** a. **B cells were isolated by magnetic sorting using CD19 microbeads from the spleens of the same pairs of young and old mice shown in Fig. 1. B cells were left unstimulated, the mRNA was extracted and qPCRperformed to evaluate RNA expression of SASP markers. Mean comparisons between groups were performed by unpaired Student’s t test (two-tailed). **b.** RNA expression of the same SASP markers in A in unstimulated FO and ABCs sorted from the spleens of young and old mice. **c.** RNA expression of Prdm1, Tbx21, Igtax, CD86, Kifc3, Syntaxin3 in unstimulated FO and ABCs sorted from the same mice in (**b**). Mean comparisons between groups were performed by two-way ANOVA. *p<0.05, **p<0.01, ***p<0.001, ****p<0.0001

We have previously shown that inflammaging induces intrinsic B cell inflammation, measured by higher expression of TNF mRNA and protein, in total B cells from old mice as compared to those from young mice [2]. Here, we compared intrinsic B cell inflammation in unstimulated total B cells isolated by magnetic sorting from the spleen of young and old mice, as well as in unstimulated FO and ABCs B cell subsets sorted from the spleen of the same mice. We measured RNA expression of several pro-inflammatory markers, many of which are markers of the senescence-associated secretory phenotype (SASP). We measured RNA for pro-inflammatory cytokines (TNF, IL-6), pro-inflammatory micro-RNAs (miRs, miR-155, miR-16), for the cell cycle regulators p16^INK4^ and p21^Waf1^ associated with cell senescence. These markers were selected because they were found negatively associated with class switch and secretion of influenza vaccine-specific antibodies in our previously published mouse [2, 23] and human [3, 24–26] B cell studies. Results in Fig. 2a show that the levels of expression of the pro-inflammatory cytokines TNF and IL-6 (top), of the pro-inflammatory miR-155 and miR-16 (center) and of the cell cycle regulators p16^INK4^ and p21^Waf1^ (bottom) are significantly higher in unstimulated B cells from old as compared to B cells from young mice.

Results in Fig. 2b show RNA expression of the markers above in sorted FO and ABCs. All these markers were found expressed at higher levels in ABCs versus FO, and higher in ABCs from old mice as compared to ABCs from young mice. These results demonstrate that ABCs are the major contributor to the pro-inflammatory phenotype of splenic B cells of old mice.

We next measured in sorted FO and ABCs RNA expression of markers described to be differentially expressed in these 2 subsets by previously published transcriptome analyses [21, 27], also confirmed by us for some of these markers evaluated in the spleens of old mice [28]. We measured those most differentially expressed in FO versus ABCs, 4 of which are associated with B cell function (Prdm1 [29], Tbx21 [30], Igtax [27, 31, 32] and CD86 [33]), whereas Kifc3 and Syntaxin3 are not [34, 35]. Results in Fig. 2c confirm and extend previously published findings that ABCs express higher levels of these markers as compared to FO B cells. These results also suggest that aging is associated with a significant increase in the expression of all these markers in ABCs, but not in FO B cells.

Our studies with human B cells have shown that higher intrinsic inflammation in unstimulated B cells from elderly individuals, as compared to B cells from young individuals, is positively associated with the secretion of IgG antibodies with autoimmune specificity [16]. This occurs because B cells from elderly individuals are already pre-activated, a status leading to spontaneous secretion of autoimmune antibodies, also observed in autoimmune diseases [17], and in the obese adipose tissue [18, 19].

### Total B cells and ABCs from old mice secrete higher amounts of IgG antibodies with autoimmune specificities as compared to those from young mice

**Fig. 3:**
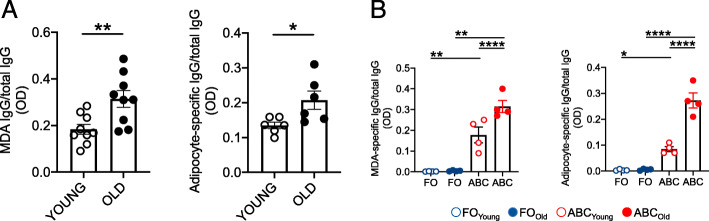
Total B cells and ABCs from old mice secrete higher amounts of IgG antibodies with autoimmune specificities as compared to those from young mice.** a. **B cells were stimulated for 7 days with CpG, then the supernatants were collected and IgG antibodies specific for MDA (left) and for adipocyte-derived antigens (right) were measured by ELISA. Results show OD of MDA-specific IgG/total IgG and adipocyte-specific IgG/total IgG, respectively. Mean comparisons between groups were performed by unpaired Student’s t test (two-tailed). **b.** IgG secretion in cultures of CpG-stimulated FO and ABCs sorted from the spleens of young and old mice. Results are expressed as ratios of MDA-specific/total IgG (left) and adipocyte-specific IgG/total IgG (right). Mean comparisons between groups were performed by two-way ANOVA. *p<0.05, **p<0.01, ****p<0.0001

We measured the secretion of IgG antibodies with autoimmune specificities in CpG-stimulated cultures of B cells from young and old mice, and we measured anti-MDA and anti-adipose tissue-derived IgG. We selected these specificites because aging is associated with increased oxidative stress and lipid peroxidation (measured by MDA) [36, 37], as well as increased fat mass (measured by adipose tissue-associated antigens) [19]. Adipose tissue-derived antigens are released from cells dying in the adipose tissue due to several mechanisms that we have previously identified, including hypoxia and cell cytotoxicity [19]. The same B cells from young and old mice in Fig. 2a were stimulated for 7 days with CpG and the culture supernatants were tested for the secretion of anti-MDA and anti-adipose tissue-derived IgG. Results in Fig. 3a expressed as normalized values (ratios MDA-specific/total IgG and adipocyte-specific IgG/total IgG), show increased secretion of autoimmune antibodies in cultures of B cells from old as compared to those from young mice.

We also tested the secretion of autoimmune antibodies in FO and ABCs sorted from the spleens of young and old mice. Results in Fig. 3b show that only ABCs and not FO B cells are able to secrete antibodies specific for MDA and for adipose tissue-derived antigens. The results in Fig. 3 altogether confirm previously published findings obtained by us [28] and by others [20, 21] showing that CpG stimulates the secretion of IgG with autoimmune specificities in B cells from young, and to a significantly higher extent, in B cells from old mice. ABCs are the likely source of IgG2c subclass, as we have previously shown [28].

Of particular interest for the secretion of autoimmune antibodies is the observed increase in Tbx21 (T-bet) and Igtax (CD11c) (Fig. 2c). It is known that ABCs originate from mature B cell subsets (FO) after in vivo or in vitro stimulation of Toll-like receptors TLR7 or TLR9, with TLR agonists plus IL-21 and IFN-γ regulating T-bet expression, whereas TLR agonists plus IL-21 alone promote CD11c expression independently of T-bet [31, 38, 39]. The signaling pathways promoting the expansion of T-bet+CD11c+ ABCs and their pathogenicity in autoimmunity remain largely unknown. It has recently been suggested a role for the interferon-regulatory factor (IRF) 5 [40], as *Irf5* deficient mice have less severe symptoms of lupus autoimmunity as compared to wild-type controls [41]. In B cells, IRF5 regulates class switch, IgG2c secretion and expression of the transcription factor Blimp-1 [42]. IRF5 was found to regulate the development of autoimmunity in mice simultaneously lacking SWAP-70 and DEF6, two Rho GTPase-regulatory proteins with immunoregulatory function [43, 44].

T-bet+CD11c+ ABCs are not only involved in autoimmunity, but they are also relevant for immunity against infections as they can persist indefinitely after influenza infection [27, 31], representing the spleen-resident population of memory B cells that secrete influenza-specific neutralizing antibodies [45].

### Total B cells and ABCs from old mice are characterized by a higher metabolic profile as compared to those from young mice

B cells that are hyper-inflammatory and secrete autoimmune antibodies are pathogenic and can also induce hyper-inflammatory pathogenic T cells, as has been shown in both mice [46] and humans [47].

**Fig. 4:**
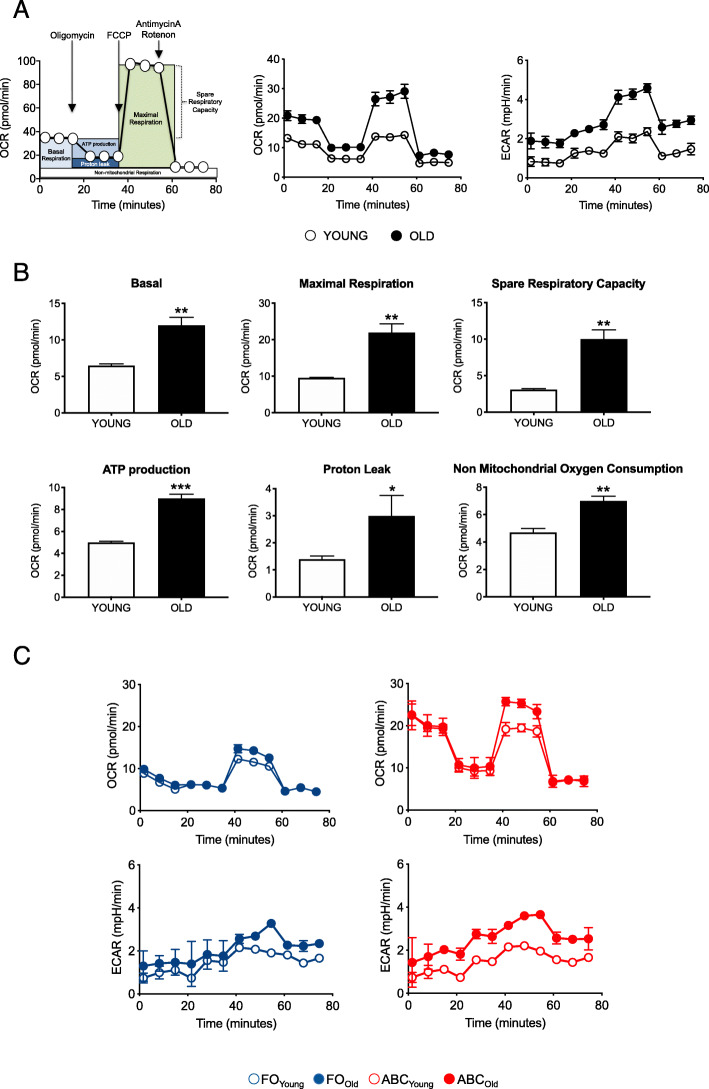
Total B cells and ABCs from old mice are characterized by a higher metabolic profile as compared to those from young mice. B cells, isolated by magnetic sorting from the spleens of young and old mice, were left unstimulated. Results are representative of 4 pairs of young and old mice. **a.** Schematic of OCR measured by a Mitostress test conducted in a Seahorse XFp extracellular flux analyzer (left). OCR results in unstimulated B cells from young and old mice (center). ECAR results in unstimulated B cells from young and old mice (right). **b.** Measures of basal respiration, maximal respiration, spare respiratory capacity, ATP production, proton leak, and non-mitochondrial respiration. Mean comparisons between groups were performed by unpaired Student’s t test (two-tailed). *p<0.05, **p<0.01, ***p<0.001. **c. **OCR (top) and ECAR (bottom) results from in unstimulated FO and ABCs sorted from the spleens of young and old mice

Substantial experimental evidences have suggested that metabolic reprogramming not only occurs but represents a crucial way to provide energy for specific cell functions, including the secretion of SASP products and of autoimmune antibodies [48–51]. Therefore, we evaluated the metabolic profile of B cells from old versus young mice. We performed a mitostress test comparing B cells from old and young mice following previously published protocols [52–54]. We seeded magnetic beads-sorted B cells from young and old mice into the wells of an extracellular flux analyzer to evaluate in real-time changes in oxygen consumption rates (OCR) and extracellular acidification rates (ECAR), measures of OXPHOS and of anaerobic glycolysis, respectively. This technology makes possible to get a variety of measures of mitochondrial function, including basal respiration, maximal respiration, spare respiratory capacity, ATP production, proton leak, and non-mitochondrial respiration with a relatively high throughput. Figure 4a (left) schematically shows the principles and the outcomes of OCR. Results in Fig. 4a (center) show higher OCR in B cells from old versus young mice. Similar to OCR, we also observed higher ECAR in B cells from old versus young mice (Fig. 4a, right). The specific measures of mitochondrial function in B cells from young and old mice are shown in Fig. 4b. In all measures, B cells from old mice show higher mitochondrial function as compared to those from young mice. These results altogether suggest that B cells from old mice have significantly higher OCR and ECAR as compared to those from young mice as they rely on a more robust glucose uptake and mitochondrial machinery as compared to those from young mice.

We also evaluated OCR and ECAR in unstimulated FO and ABCs sorted from the spleen of young and old mice. Results in Fig. 4c show that OCR (top) and ECAR (bottom) measures are higher in ABCs than in FO, and more in ABCs from old as compared to ABCs from young mice. These results demonstrate that ABCs are hyper-metabolic as compared to FO likely because ABCs need more energy to support their secretory phenotype.

**Fig. 5:**
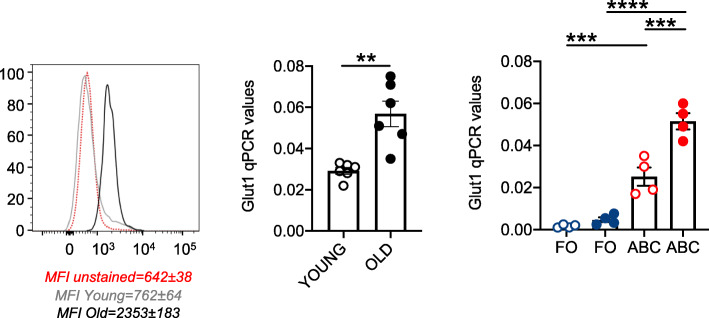
Total B cells and ABCs from old mice are characterized by higher glucose uptake as compared to those from young mice. B cells, isolated by magnetic sorting from the spleens of young and old mice, were left unstimulated. Glucose uptake was measured by flow cytometry and the glucose fluorescent analog 2-NBDG. Results show MFI (mean fluorescence intensity) from one representative experiment. MFI±SE from 4 independent experiments in which 4 mice were evaluated are shown below the histogram (**left**). The mRNA expression of the glucose transporter Glut1 was measured by qPCR (**center**). Mean comparisons between groups were performed by unpaired Student’s t test (two-tailed). Glut1 expression in unstimulated FO and ABCs sorted from the spleens of young and old mice (**right**). Mean comparisons between groups were performed by two-way ANOVA. **p<0.01, ***p<0.001, ****p<0.0001

We then measured glucose uptake in unstimulated B cells by using flow cytometry and the glucose fluorescent analog 2-NBDG, following a previously published protocol [55], and found increased glucose uptake Fig. 5 (left) and increased mRNA expression of the glucose transporter Glut1, the main receptor for glucose uptake in B cells as shown in human studies [56, 57] (center), in unstimulated B cells from old versus young mice. When we measured Glut1 expression in unstimulated sorted B cell subsets, we found higher levels of expression in ABCs than in FO, confirming the hyper-metabolic profile of ABCs. Again, the levels were higher in ABCs from old as compared to ABCs from young mice (right).

### Total B cells and ABCs from old mice are characterized by higher mRNA expression of enzymes involved in metabolic pathways as compared to those from young mice

**Fig. 6:**
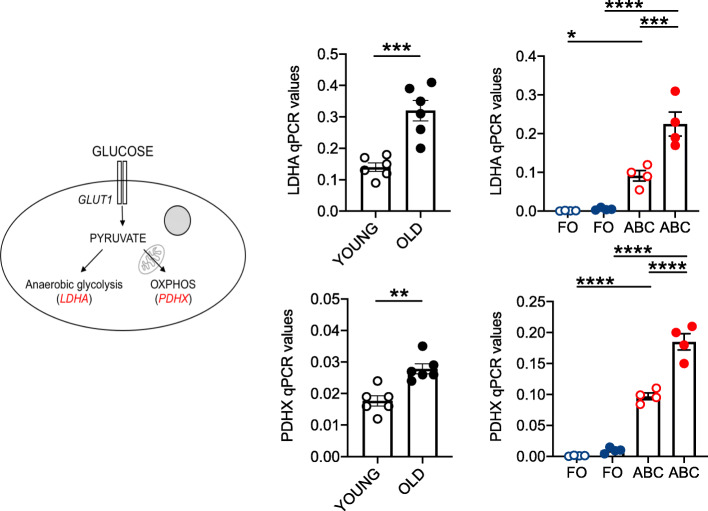
Total B cells and ABCs from old mice are characterized by higher mRNA expression of enzymes involved in metabolic pathways as compared to those from young mice.** Left. **A diagram depicting the two major pathways analyzed is shown, with the enzymes used to measure anaerobic glycolysis (LDHA) and OXPHOS (PDHX) in parentheses.** Center. **B cells, isolated by magnetic sorting from the spleens of young and old mice, were left unstimulated, the mRNA was extracted and qPCRperformed to evaluate RNA expression of LDHA (**top**) and PDHX (**bottom**). Mean comparisons between groups were performed by unpaired Student’s t test (two-tailed). **Right**. RNA expression of LDHA (**top**) and PDHX (**bottom**) in unstimulated FO and ABCs sorted from the spleens of young and old mice. Mean comparisons between groups were performed by two-way ANOVA. *p<0.05, **p<0.01, ***p<0.001, ****p<0.0001

To confirm the results on the metabolic profile of unstimulated B cells from young and old mice, we also measured mRNA expression of genes encoding enzymes associated with cell metabolism. We measured by qPCR mRNA levels of pyruvate dehydrogenase (PDHX) as a measure of OXPHOS [58, 59], and mRNA levels of lactate dehydrogenase (LDHA) as a measure of glycolysis [60–62]. Figure 6 (left) shows the pathways downstream of glucose uptake, leading to the generation of cytoplasmic pyruvate which is processed through OXPHOS by PDHX or through anaerobic glycolysis by LDHA. Results in Fig. 6 (center) show that unstimulated B cells from old mice have higher levels of transcripts of PDHX and LDHA, as compared to B cells from young mice, confirming OCR and ECAR results, respectively. This indicates that B cells from old mice convert pyruvate into both lactate and acetyl-CoA for entrance into the Krebs cycle more efficiently than B cells from young mice. Results in Fig. 6 (right) show that ABCs express higher levels of transcripts for both LDHA and PDHX enzymes than FO, in support of their hyper-metabolic profile. The results in Figs. 5 and 6 altogether confirm the hyper-metabolic profile of B cells from old mice, as compared to those from young mice, which is associated with higher expression of SASP markers and higher secretion of antibodies with autoimmune reactivity.

## Conclusions

Aging is associated with increased inflammaging and intrinsic B cell inflammation, decreased protective antibody responses and increased autoimmune antibody responses. Results herein have shown that the of B cells from old mice before stimulation express higher levels of RNA for pro-inflammatory markers which is associated with higher secretion of IgG antibodies specific for MDA and for adipocyte-derived antigens. These hyper-inflammatory B cells from old mice also perform increased glycolysis and OXPHOS, as compared to B cells from young mice, a feature needed to support their higher secretory profile (pro-inflammatory products and autoimmune antibodies), as we have recently shown in humans [16]. Results herein have also identified ABCs, whose frequencies and numbers increase and are the most pro-inflammatory B cell subset, as the cell type responsible for the expression of inflammatory markers and secretion of autoimmune antibodies in the spleen of old mice. Our results allow the identification of a relationship between intrinsic inflammation, metabolism and autoimmune B cells, advancing our understanding of critical mechanisms leading to the generation of pathogenic B cells. Pathogenic B cells that are hyper-inflammatory and secrete autoimmune antibodies can also induce pro-inflammatory T cells in both mice [46] and humans [47], and it has been shown that immunotherapy of autoimmune (Rheumatoid Arthritis) patients with anti-CD20 antibody not only specifically depletes B cells, but also blocks glucose uptake and usage in T cells and impairs the differentiation of pathogenic T cells, leading to an improved health condition.

## Methods

### Mice

Male C57BL/6 mice, both young (3–4 months) and old (18–22 months), were obtained from the National Institutes on Aging and maintained in our AAALAC-certified facility. Mice were acclimated for at least 7 days before sacrifice. Mice with evidence of disease, mainly tumors, skin and eye infections, were not used in these studies. Mice were allowed to freely access food and water and were housed at 23°C on a 12 hr light/dark cycle. All studies adhered to the principles of laboratory animal care guidelines and were IACUC approved (protocol # 19-199-LF).

### Flow cytometry

Splenic B cells were membrane stained for 20 min at room temperature with Live/Dead fixable stain (ThermoFisher) and with the following antibodies: PerCP-conjugated anti-CD45 (Biolegend 103,130), APC-Cy7-conjugated anti-CD19 (BD 557,655), PE-Cy7-conjugated anti-AA4.1 (eBioscience 25-5892-81), APC-conjugated anti-CD43 (BD 560,663), FITC-conjugated anti-CD21/CD35 (BD 553,818) and PE-conjugated anti-CD23 (BD 553,139), to identify Follicular (FO, CD19+AA4.1/CD43-CD23+CD21-), Marginal Zone (MZ, CD19+, AA4.1/CD43-CD23-CD21+) and Age-associated B cells (ABCs, CD19+AA4.1/CD43-CD23-CD21-) B cell subsets. AA4.1 (CD93) is the marker of transitional B cells. CD43 is the marker of B1 B cells. Both transitional and B1 B cells are excluded by cell staining. Cells were then fixed with BD Cytofix (BD 554,655). Up to 10^6^ events in the lymphocyte gate were acquired on an LSR-Fortessa (BD) and analyzed using FlowJo 10.5.3 software.

### B cell sorting

B cells were isolated from dissociated spleens after 20 min incubation at 4°C using CD19 MicroBeads (Miltenyi Biotec 130-121-301), according to the MiniMACS protocol (20 µl Microbeads + 80 µl PBS, for 10^7^ cells). At the end of the purification procedure, cells were 90–95 % CD19-positive by cytofluorimetric analysis. Within 15 minutes of magnetic sorting, B cells were divided into 3 aliquots: one immediately resuspended in TRIzol (ThermoFisher Scientific), one used for culture setup within 2 hrs, and one used for Seahorse experiments within 1 hr.

FO and ABC B cell subsets were sorted at the Sony SH800 cell sorter using the markers above.

### B cell culture

Same numbers of total B cells, FO and ABCs, from young and old mice were left unstimulated or were cultured at the concentration of 10^6^/ml in complete medium (RPMI 1640, supplemented with 10 % FCS, 100 U/ml Penicillin-Streptomycin, 2 × 10^− 5^ M 2-ME, and 2 mM L-glutamine). FCS was certified to be endotoxin-free. Cells were stimulated in 24-well culture plates with 5 µg/ml of CpG (invivoGen ODN2006) for 7 days (to measure IgG secretion in culture supernatants). At the end of the stimulation time, cells were counted in a solution of trypan blue to evaluate viability which was found comparable in cultures of young and old mice.

### Quantitative PCR (qPCR)

To evaluate RNA expression of enzymes involved in metabolic pathways, the mRNA was extracted from same numbers of unstimulated total B cells, or unstimulated FO and ABCs, from young and old mice, using the µMACS mRNA isolation kit (Miltenyi), according to the manufacturer’s protocol, eluted into 75 µl of preheated elution buffer, and stored at -80°C until use.

To evaluate RNA expression of pro-inflammatory markers, same numbers of unstimulated total B cells, or unstimulated FO and ABCs, from young and old mice, were resuspended in TRIzol, according to the manufacturer’s protocol, resuspended in 10 µl of preheated H_2_O, and stored at -80°C until use.

Reverse Transcriptase (RT) reactions were performed in a Mastercycler Eppendorf Thermocycler to obtain cDNA. Briefly, 10 µl of mRNA or 2 µl of RNA at the concentration of 0.5 µg/µl were used as template for cDNA synthesis in the RT reaction. Conditions were: 40 minutes at 42°C and 5 minutes at 65°C. For miRs quantification, RT reactions were performed in the presence of specific primers. qPCR reactions were conducted in MicroAmp 96-well plates, and run in the ABI 7300 machine. Calculations were made with ABI software. Briefly, we determined the cycle number at which transcripts reached a significant threshold (Ct). A value for the amount of the target gene, relative to GAPDH for mRNAs or to U6 for miRs, was calculated and expressed as ΔCt. Reagents and primers for qPCR amplification were from ThermoFisher.

### Enzyme‐Linked Immunosorbent Assay (ELISA)

To measure total IgG antibodies, ELISA plates were coated with purified goat anti-mouse IgG (Jackson ImmunoResearch 115-005-062) at the concentration of 2 µg/ml.

To measure IgG antibodies with autoimmune specificity, ELISA plates were coated with Malondealdehyde (MDA) or with adipocyte-derived antigens, both at the concentration of 10 µg/ml.

To measure IgG antibodies specific for adipocyte-derived antigens, ELISA plates were coated with protein lysates of adipocytes isolated from mouse epidydymal fat pads as previously described [28]. Briefly, The adipose tissue was harvested, weighed and washed with 1X Hanks’ Balanced salt Solution (HBSS). It was then resuspended in Dulbecco’s modified Eagle’s Medium (DMEM), minced into small pieces, passed through a 70 µm filter and digested with collagenase type I (SIGMA C-9263) for 1 hr in a 37°C water bath. Digested cells were passed through a 300 µm filter, centrifuged at 300 g in order to separate the floating adipocytes from the stromal vascular fraction containing the immune cells. The cells floating on the top were transferred to a new tube as adipocytes and washed 3 times with DMEM in a 5415 C Eppendorf microfuge (2,000 rpm, 5 min). To obtain cytoplasmic protein extracts, the adipocytes (100 µl) were lysed resuspended in 50 µl of a lysing solution (Hepes 10 mM, pH 7.9; KCl 10 mM; EDTA 1.0 mM; MgCl_2_ 1.5 mM; 1mM Na_3_VO_4_; Nonidet P-40 (0.1 %) and protease inhibitors), vortexed and centrifuged at 8,000 rpm for 5 min at 4°C. Aliquots of the cytoplasmic protein extracts were stored at -80°C. Protein content was determined by Bradford [63].

Detection antibody was a biotinylated anti-IgG antibody, followed by streptavidin-HRP.

### Metabolic measurements

OCR and ECAR were measured in a Mitostress test conducted in a Seahorse XFp extracellular flux analyzer (Agilent). Briefly, same numbers of splenic B cells, and FO and ABCs, from young and old mice were seeded in a CellTAK (BD Biosciences)-coated plate. Cells, at the concentration of 2.5 × 10^5^/well, were initially incubated in XF DMEM medium supplemented with glutamine, glucose and pyruvate (200 µL of each reagent in 20 mL of medium). Maximal respiratory capacity was measured by treating with Oligomycin (1 µM) to block ATP production, followed by the uncoupling agent FCCP (fluoro-carbonyl cyanide phenylhydrazone, 5 µM), to dissipate proton gradients and allow electron transport and oxygen consumption to operate at maximal rate. This elevated OCR is suppressed by Rotenone/Antimycin (1 µM), showing that respiration is mitochondrial. To confirm Seahorse results, we also analyzed the metabolic status of splenic B cells by qPCR gene expression analysis of: Glucose transporter 1 (Glut1), that facilitates the transport of glucose across the plasma membrane; LDHA (Lactate Dehydrogenase) involved in anaerobic glycolysis that converts pyruvate into lactate; PDHX (Pyruvate Dehydrogenase), involved in oxidative phosphorylation, a component of the pyruvate dehydrogenase complex, which converts pyruvate into acetyl-CoA, used in the Kreb cycle.

### Glucose uptake measurement

To measure glucose uptake, unstimulated B cells from young and old mice (10^6^/mL) were stained with the fluorescent glucose analog (2-(N-(7-Nitrobenz-2-oxa-1,3-diazol-4-yl)Amino)-2-Deoxyglucose) (2-NBDG, Thermo Fisher N13195) that was added at a final concentration of 50 µM for 30 min. Cells were then washed and immediately acquired an LSR-Fortessa (BD), using the FITC channel to detect the signal from the fluorescent glucose uptake tracker. Fluorescence data were analyzed using FlowJo 10.5.3 software.

### Statistical analyses

To examine differences between groups, Student’s t tests (two-tailed) were used. To examine differences between 4 groups, two-way ANOVA was used. Group-wise differences were analyzed afterwards with Bonferroni’s multiple comparisons test, with *p* < 0.05 set as criterion for significance. To examine differences between 2 groups, Student’s t tests (two-tailed) were used. Analyses were performed using GraphPad Prism 8.4.3 software.

## Data Availability

Not applicable.

## Abbreviations

ABCs: Age-associated B cells
ECAR: Extracellular acidification rate
MDA: Malondialdehyde
2-NBDG: 2-(N-(7-Nitrobenz-2-oxa-1,3-diazol-4-yl)Amino)-2-Deoxyglucose
OCR: Oxygen consumption rate
SASP: Senescence-associated secretory phenotype
